# Optimization of a Modular Nanotransporter Design for Targeted Intracellular Delivery of Photosensitizer

**DOI:** 10.3390/pharmaceutics16081083

**Published:** 2024-08-18

**Authors:** Rena T. Alieva, Alexey V. Ulasov, Yuri V. Khramtsov, Tatiana A. Slastnikova, Tatiana N. Lupanova, Maria A. Gribova, Georgii P. Georgiev, Andrey A. Rosenkranz

**Affiliations:** 1 Laboratory of Molecular Genetics of Intracellular Transport, Institute of Gene Biology of Russian Academy of Sciences, 34/5 Vavilov St., 119334 Moscow, Russia; 2 Faculty of Biology, Lomonosov Moscow State University, 1-12 Leninskie Gory St., 119234 Moscow, Russia

**Keywords:** modular nanotransporters, targeted drug delivery, photosensitizers, anticancer drugs, photodynamic effect, epidermal growth factor receptors, binding, importins, Keap1

## Abstract

Modular nanotransporters (MNTs) are drug delivery systems for targeted cancer treatment. As MNTs are composed of several modules, they offer the advantage of high specificity and biocompatibility in delivering drugs to the target compartment of cancer cells. The large carrier module brings together functioning MNT modules and serves as a platform for drug attachment. The development of smaller-sized MNTs via truncation of the carrier module appears advantageous in facilitating tissue penetration. In this study, two new MNTs with a truncated carrier module containing either an N-terminal (MNT_N_) or a C-terminal (MNT_C_) part were developed by genetic engineering. Both new MNTs demonstrated a high affinity for target receptors, as revealed by fluorescent-labeled ligand-competitive binding. The liposome leakage assay proved the endosomolytic activity of MNTs. Binding to the importin heterodimer of each truncated MNT was revealed by a thermophoresis assay, while only MNT_N_ possessed binding to Keap1. Finally, the photodynamic efficacy of the photosensitizer attached to MNT_N_ was significantly higher than when attached to either MNT_C_ or the original MNTs. Thus, this work reveals that MNT’s carrier module can be truncated without losing MNT functionality, favoring the N-terminal part of the carrier module due to its ability to bind Keap1.

## 1. Introduction

One of the most essential factors in effective cancer treatment is the treatment’s ability to act specifically on tumor cells [1]. Therefore, the development of targeted drug delivery systems represents a promising area for obtaining more effective anticancer treatment modalities [2]. Polypeptide constructs as drug carriers for targeting intracellular sites are advantageous due to their high specificity and biocompatibility. However, they often face challenges such as rapid degradation and low bioavailability, mainly because of the necessity to penetrate lipid membranes [3,4,5,6]. One of these systems is modular nanotransporters (MNTs), which are artificial polypeptides composed of several transport modules [7]. MNTs are capable of increasing the efficiency of the delivery of cytotoxic agents into the nuclei of cancer target cells, which greatly enhances the effectiveness of photodynamic therapy (PDT) [8,9] and radionuclide therapy [10,11]. PDT is an emerging treatment method for skin neoplasms and a number of solid tumors, including esophagus, lung, and prostate cancers [12,13,14]. PDT employs substances that can transit to excited triplet states in response to light, producing reactive oxygen species (ROS), mainly singlet oxygen, that cause biomolecular damage, activation of the immune response, and cell death [13,15,16]. ROS with short lifetimes induce damage in the nanometer range from their source [17,18,19], resulting in the spatial restriction of the effect within the cell. Therefore, intracellular delivery to the most damage-sensitive structures of cancer cells is necessary for maximum efficiency. Previous studies have demonstrated that delivering photosensitizers to the cancer cell nuclei via MNTs can increase their photodynamic activity by up to 2500 times [20]. It is unclear, however, how delivery to other photosensitizer-sensitive cell compartments will impact the effectiveness of photodamage. The mitochondria are examples of photosensitive cellular compartments [21,22,23].

**Figure 1:**
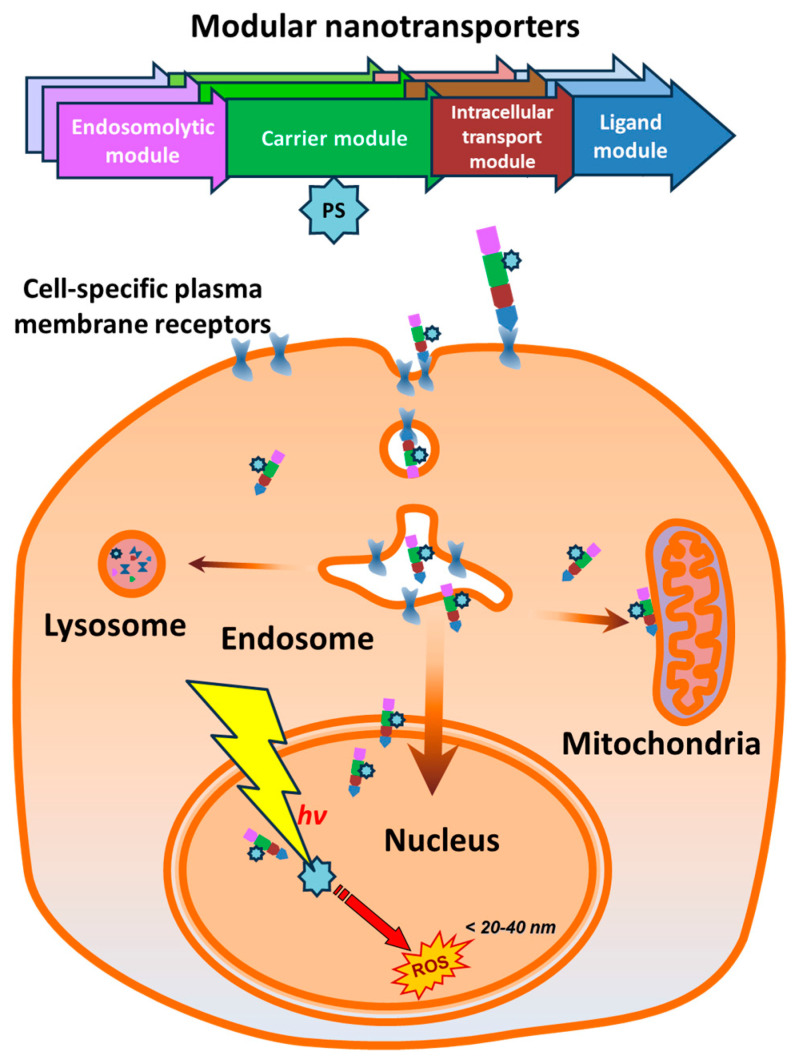
Principal scheme of modular nanotransporter-mediated targeted intracellular delivery of photosensitizers (PS). Modular nanotransporters recognize target cells binding to cell-specific plasma membrane receptors via a ligand module. Following receptor internalization, modular nanotransporters exit endosomes with the help of an endosomolytic module and then can deliver their cargo to the target intracellular compartment (e.g., cell nucleus). PS—photosensitizer; ROS—reactive oxygen species.

The use of multifunctional macromolecules and nanoparticles makes it possible to create constructs for the delivery of drugs to a suitable compartment of target cells [24,25,26]. However, the transport of macromolecules into the specified cells and between the cell compartments is controlled by numerous regulatory mechanisms [27]. The above-mentioned MNTs are created for utilizing these mechanisms (Figure 1). Each module of MNT provides a particular stage of transport when the MNT enters the target cell and then relocates to a specified cell compartment. MNTs, unlike many other therapeutic polypeptides, can permeate membranes and reach the cytosol of target cells. The sequences of MNT modules derived from natural proteins are united in a single gene sequence that encodes a chimeric protein capable of delivering drugs to a specified location of the target cell. MNT modules are interchangeable, allowing for customization based on the target cell type and the desired intracellular location for drug delivery [7]. Depending on their composition, MNTs can penetrate, for example, cell nuclei, lysosomes, or bind to the surface of mitochondria [7,11,20,28]. They contain a ligand module that binds to overexpressed cell surface receptors, allowing it to recognize a specified cell type. Receptor-mediated endocytosis leads to the internalization of MNT, and for subsequent release from endosomes, MNTs contain an endosomolytic module capable of destroying lipid membranes in a slightly acidic environment within endosomes. MNTs can also contain modules for transport to a specific cellular compartment, most commonly a nuclear localization signal (NLS) for the delivery of drugs into the nucleus [7]. The carrier module combines functionally active MNT modules and is also used to attach drugs that are transported by the transporter. Most of the MNT constructs developed to date use the hemoglobin-like protein (HMP, [29]) from *Escherichia coli* as the carrier module. Reducing the size of macromolecules facilitates the diffusion in tissues, which contributes to a greater depth of penetration and, consequently, better delivery of the active principle [30]. HMP performs the function of a carrier and does not play a significant role in interaction with cellular structures during transport into the target cell compartments. Therefore, in order to reduce the size of MNTs, it may be reasonable to consider truncating this module. HMP has two domains: the beta-folded C-terminal domain and the alpha-helical N-terminal globin domain with a pocket for porphyrin [30]. Keeping one of these HMP domains as a carrier module allows us to create a smaller construct. Because a crucial function of the carrier module is to ensure the availability of MNTs’ endosomolytic and ligand modules for interaction with cellular structures, it requires truncation without compromising their activity.

Another rationale for the development of constructs with a truncated carrier module is the recently discovered interaction of HMP, which is part of one of the MNTs, with Kelch-like ECH-associated protein 1 (Keap1) [28]. Keap1 is widely represented in the cytoplasm and is involved in the control of many cellular activities, primarily the regulation of the level of transcription factor NF-E2-related factor 2 (Nrf2) [31,32,33]. In addition, Keap1 interacts with the PGAM5 protein, which is localized on the outer membrane of mitochondria and is also involved in maintaining mitochondrial homeostasis [34,35]. Interaction with Keap1 can lead to the retention of MNTs in the cytoplasm and the activation of cell signaling pathways, resulting in defense from the oxidative process. On the other hand, through the interaction of MNTs with Keap1, it is possible to target cytotoxic agents to mitochondria. As demonstrated in our recent work, attaching a photosensitizer to MNTs with the anti-Keap1 monobody sequence increases the photocytotoxicity compared to a photosensitizer attached to cytosol-targeted MNTs [28]. Thus, the development and study of new MNTs will shed light on the possibilities of creating functional MNT structures in smaller sizes. Furthermore, this allows us to examine the effect of an additional mitochondrial localization site on the photocytotoxicity of photosensitizers delivered to the nucleus of a cancer target cell.

## 2. Materials and Methods

### 2.1. Cell Lines

The human epidermoid carcinoma A431 cell line was obtained from the American Type Culture Collection (ATCC, Manassas, VA, USA) and maintained according to the ATCC specification.

### 2.2. Producing MNTs with a Truncated Carrier Module

To create MNTs with a truncated carrier module, the previously described construct (DTox-HMP-NLS-EGF) was used. The MNTs consist of the sequence of human epidermal growth factor (EGF) as a ligand module. The diphtheria toxin translocation domain (DTox) sequence was employed as an endosomolytic module. The sequence of the hemoglobin-like HMP protein was taken as a carrier module. The optimized nuclear localization signal (NLS) of the large T-antigen of the SV40 virus was used as a module for delivery into the cell nucleus. We obtained two MNT mutants with C- or N-terminal parts of the HMP module. Mutagenesis was performed using oligonucleotide primers by PCR amplification of the original gene without the corresponding fragments. The primers were synthesized by Evrogen, Moscow, Russia, and mutagenesis was performed using the QuickChange™ kit (Agilent Technologies, Santa Clara, CA, USA). Competent cells (XL1-Gold) were transformed with the obtained plasmids and grown in a super optimal broth and catabolic repressor (SOC) medium for one hour at 37 °C in the presence of ampicillin. Colonies with mutations were selected by PCR screening according to a standard protocol [36]. Furthermore, restriction analysis was used to determine whether a deletion happened in the areas of interest [37]. The obtained plasmids were sequenced using the Sanger technique [38].

### 2.3. MNT Expression and Purification

The production of DTox-HMP_C_-NLS-EGF (MNT_C_) with the C-terminal fragment of HMP, DTox-HMP_N_-NLS-EGF (MNT_N_) with the N-terminal fragment of HMP, and the original MNT (DTox-HMP-NLS-EGF, hereinafter referred to as the MNT_F_) was carried out in the strain *E. coli* BL21(DE3). The cells were grown on LB Broth Miller medium (Amresco, Solon, OH, USA). The induction of MNT expression was performed with 200 µM of isopropyl-β-D-galactopyranoside (SybEnzyme, SybEnzyme, Russia) for 2.5 h at 18 °C. All MNTs were isolated from the soluble fraction and purified by affinity chromatography on a HisTrap™ high-performance carrier (Cytiva, Muskegon, MI, USA). Purified MNTs were refolded in 150 mM NaCl, 25 mM Na_2_HPO_4_, 0.5 mM phenylmethylsulfonyl fluoride, 1 mM EDTA, 2 mM oxidized glutathione, and 0.67 mM reduced glutathione. After refolding, the MNTs were re-purified by affinity chromatography and dialyzed against PBS (10 mM Na_2_HPO_4_, 150 mM NaCl, pH 8). The purity of MNTs produced using the given approach was approximately 85%, according to denaturing electrophoresis data (Figure S1). The concentration of MNTs was assessed using the Bradford method [39].

### 2.4. Binding Assay

The effectiveness of the interaction of the ligand module with the epidermal growth factor receptor (EGFR) was evaluated on the cell line of human epidermal carcinoma A431, characterized by overexpression of EGFR. To achieve this, we determined the dissociation constant of the ligand–receptor complex in competition with a fluorescent-labeled MNT_1_ [28]. MNT_1_ was labeled with Alexa-647, as described earlier [40]. In brief, a freshly prepared solution of Alexa Fluor 647 succinimidyl ester (Molecular Probes, Eugene, OR, USA) (2 mg/mL) was added to the MNT_1_ solution in carbonate buffer, a pH of 8.6 in molar excess of 5:1. Labeled MNT_1_ was purified by ultrafiltration. Binding analysis was performed on A431 cells, which were dispersed onto 48-well plates and fixed with a 0.5% paraformaldehyde solution for 5 min on ice. Incubation with MNTs was performed in Dulbecco’s Modified Eagle medium (DMEM) containing 1% bovine serum albumin (BSA), 100 μg/mL gentamicin, 0.4 g/L NaHCO_3_, 15 mM HEPES, a pH of 7.3, overnight at +4 °C. For the study of competitive binding, 25 nM of labeled MNT_1_ and different concentrations of MNT_C_ or MNT_N_ were used. After incubation, the cells were washed five times with Hanks solution, and 100 µL of 0.25% trypsin-EDTA solution per well was added for 2 h. The well contents were removed to a black-well plate, and the wells were rinsed with 150 µL of a 1% Triton X-100 solution. Sample fluorescence was measured in the 630–680 nm range using a ClarioSTAR Plus plate reader (BMG LABTECH, Ortenberg, Germany) with an excitation wavelength of 625–630 nm.

### 2.5. Liposome Leakage Assay

The ability of MNT_1_ to provide liposome leakage was demonstrated on small unilamellar phosphatidylcholine (Sigma-Aldrich, Burlington, MA, USA) liposomes prepared by reverse-phase evaporation [41] loaded with the fluorescent dye calcein (Fluka, Seelze, Germany) to a concentration of 100 mM, which led to fluorescence self-quenching. Liposome leakage analysis was performed as described previously [28].

### 2.6. Thermophoresis

Binding between Keap1 and MNTs was assessed using a Monolith NT.115 instrument (NanoTemper Technologies, München, Germany) in phosphate buffer containing sodium phosphate (25 mM) and sodium chloride (150 mM), a pH of 8.0, using Cy3-labeled Keap1, as described in our previous article [28].

The interaction of the MNTs with the α/β importin complex was evaluated in a buffer containing 20 mM HEPES, 110 mM KCl, 5 mM NaHCO_3_, 5 mM MgCl_2_, 0.1 mM CaCl_2_, 1 mM EGTA, and 1 mM DTT, a pH of 7.4, using Cy3-labeled β-importin as described earlier [42].

### 2.7. Photocytotoxicity

Chlorin *e*_6_ (Frontier Scientific, Philadelphia, PA, USA) was coupled to the MNTs using 1-ethyl-3-(3-dimethylaminopropyl)-carbodiimide and N-hydroxysuccinimide (Sigma-Aldrich) as described previously [28]. Briefly, a 3 mM aqueous solution of sodium chlorin *e*_6_ was diluted in a buffer containing 10 mM MES (Sigma-Aldrich), pH 6.5, and 1 mM sodium dodecyl sulfate (SDS-Na) to a final concentration of 150 μM. The resulting solution was mixed with freshly prepared 0.1 M aqueous solutions of 1-ethyl-3-(3-dimethylaminopropyl)-carbodiimide (Sigma-Aldrich) and N-hydroxysuccinimide (Sigma-Aldrich) up to the molar ratio clorin/carbodiimide/N-hydroxysuccinimide = 1:4.5:15. The solution of the activated chlorin *e*_6_ was added to 10 μM of MNTs in a buffer containing 30 mM sodium phosphate, 20 mM sodium borate, 150 mM sodium chloride, a pH of 8.0, and SDS-Na in the molar ratio MNT:SDS-Na = 1:5. The reaction was stopped the next day with hydroxylamine (Reakhim, Moscow, Russia) at a final concentration of 10 mM. The conjugates were purified using affinity chromatography on Ni-NTA Sepharose.

A431 cells were seeded in 96-well plates (1000 cells per well) for the cytotoxicity experiment. The next day, the media was changed to DMEM with 10 mg/mL BSA, and the cells were treated with various doses of MNT-chlorin conjugates or free chlorin *e*_6_. After 20 h of incubation, the cells were washed twice with Hank’s solution and irradiated for 10 min (0.0276 W/cm^2^) with a halogen lamp using a slide projector in Hank’s solution. In order to provide a uniform (estimated deviation less than 10%) distribution of luminous power in experimental wells, we illuminated one half of the plate, followed by illumination of the second half. The non-illuminated half of the plate was shielded from the light source. Hank’s solution was then replaced with fresh medium containing 10% fetal bovine serum and incubated for 4–5 days. Cell viability was assessed using 3-(4,5-dimethylthiazol-2-yl)-2,5-diphenyltetrazolium bromide (MTT). The experiments for each MNT-chlorin e6 conjugate and free chlorin *e*_6_ were repeated six times, and each of them was performed in six replicates (one column in a plate per experimental point, excluding perimeter wells). The concentration causing 50% cell death (EC_50_) was estimated using interpolation of data along a four-parameter logistic curve, as is usually used for the analysis of photocytotoxicity on cell cultures [43,44,45], in the GraphPad Prism 9.0 software package (GraphPad Software Inc., La Jolla, CA, USA). The significance of the differences was assessed using ANOVA with the GraphPad Prism 9.0 software.

## 3. Results

For this study, plasmids encoding MNTs with C-terminal and N-terminal parts of the carrier module were obtained. Both truncated MNTs were successfully produced and purified, as described in the “Materials and Methods” section.

**Figure 2:**
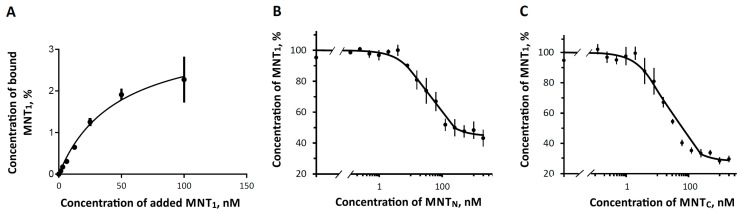
Binding of MNTs to EGFR of A431 cells. (**A**) Binding of Alexa647-MNT_1_ to A431 cells; (**B**) and (**C**) represent concurrent binding of Alexa647-MNT_1_ to MNT_N_ and MNT_C_, respectively. The data of typical experiments performed in three repetitions is presented. The data are presented as average values ± SEM.

A study of the binding of fluorescently labeled MNT_1_ showed that it binds to EGFR on the surface of A431 cells with a dissociation constant, K_d_ 46 ± 15 nM (mean ± SEM) (Figure 2A). This value is consistent with the data obtained by radioligand analysis (62 ± 10 nM) [28]. Competitive analysis yielded K_d_ values of 10 ± 2 nM for MNT_C_ and 20 ± 4 nM for MNT_N_ (Figure 2B,C). This demonstrates that the ligand module in both MNTs retains functionality and can target MNT cells with overexpressed EGFR.

**Figure 3:**
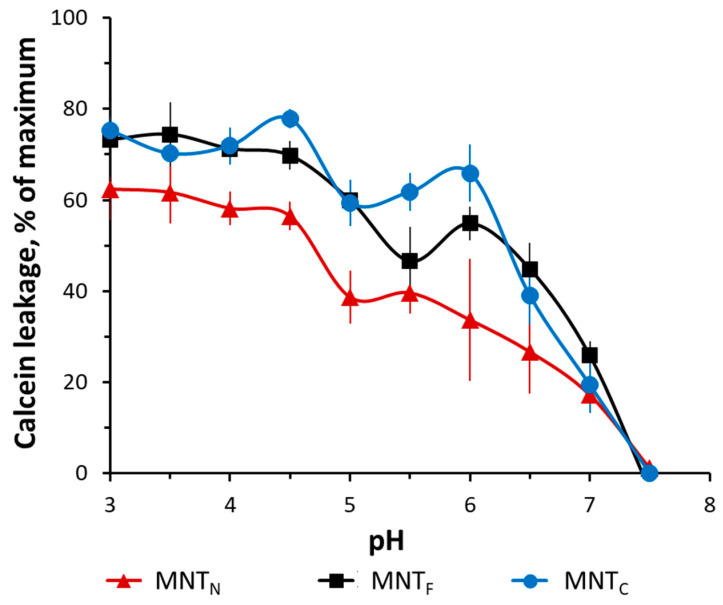
MNT-induced dye leakage from phosphatidylcholine liposomes loaded with the fluorescent dye calcein to the fluorescence self-quenching concentration. The data are mean values ± SEM.

Figure 3 shows the leakage of the fluorescent dye calcein from phosphatidylcholine liposomes at different pH levels. The experiments have shown that the new MNTs have membranolytic activity in the pH 5–6 region corresponding to the pH of the endosomes, which makes it possible to release MNTs from early endosomes after internalization.

Presumably, HMP participates in the membranolytic activity of MNTs, and its truncation could lead to a decrease in this activity in the area of slightly acidic pH. As shown in Figure 3, MNTs containing the N-terminal HMP domain exhibit somewhat less activity in this range. At a pH of 6, MNT_N_ releases approximately 45% of calcein, while MNTs with full-size HMP release nearly 60%. Despite this, it appears that the loss of one of the HMP domains has no significant impact on MNT–membrane interaction.

The thermophoresis assay enables the observation of a change in the diffusion and thermodiffusion of molecules during the formation of complexes by registering fluorescence from one of them [46]. For this purpose, a laser is used to heat the capillary containing the sample, resulting in the formation of a temperature gradient along which the molecules move. The interaction between molecules and the formation of their complexes influences the speed of movement of the labeled molecules. As a result, the change in concentration of the examined substance causes a change in fluorescence at the given time point of the thermophoretic curve. The interaction constant of the examined macromolecules can be determined by estimating the observed fluorescence levels over concentration based on theoretical dependencies.

**Figure 4:**
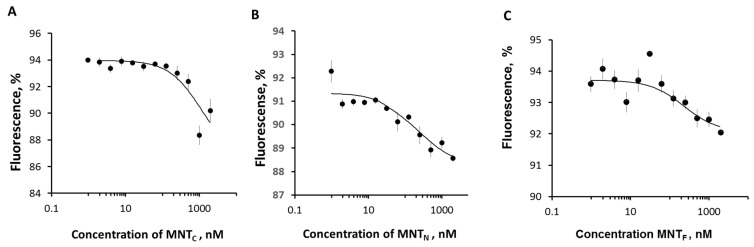
The interaction of MNT_S_ with Keap1-Cy3 assessed by thermophoresis. Dependences of relative fluorescence intensities (fluorescence intensity before the start of thermophoresis is taken as 100%) at 20 s after the start of thermophoresis on the concentration of the MNT_C_ (**A**), MNT_N_ (**B**), and MNT_F_ (**C**) at a constant concentration of the Keap1-Cy3. Standard errors (SEs) of relative fluorescence intensities are shown (*n* = 12).

Thermophoretic curves for the interaction of MNT_C_ and MNT_N_ with Keap1 are shown in Figure 4A and Figure 4B, respectively. The results of the experiments demonstrated that MNT_N_ with an N-terminal domain of HMP interacts with Keap1 with K_d_ of 428 ± 193 nM. At the same time, MNT_C_ has a much higher dissociation constant of the complex with Keap1 (K_d_ = 1800 ± 500 nM). The difference between the measured values of the dissociation constants is significant (*p* < 0.05, *n* = 12). In addition, the values of dissociation constants for MNT_C_ and MNT_F_ with full-size HMP (205 ± 22 nM) differ significantly (Figure 4). At the same time, the difference in dissociation constants for MNT_F_ and MNT_N_ is nonsignificant. Thus, it can be concluded that HMP interacts with Keap1 due to the N-terminal (globin) part.

**Figure 5:**
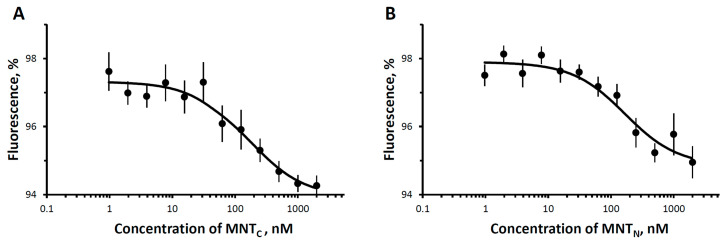
The interaction of MNTs with α/β importin complex labeled with Cy3 assessed by thermophoresis. Dependences of relative fluorescence intensities (fluorescence intensity before the start of thermophoresis is taken as 100%) at 20 s after the start of thermophoresis on the concentration of MNT_N_ (**A**) and MNT_C_ (**B**) at a constant concentration of the importin complex. Standard errors (SE) of relative fluorescence intensities are shown (*n* = 7–9).

The thermophoretic curves of the interaction of MNTs with α/β-importins are shown in Figure 5. The dissociation constants of the new constructs with the complex of imports were 160 ± 31 nM and 179 ± 22 nM for MNT_N_ and MNT_C_, respectively. This demonstrates that the modules for delivery to the nucleus of these MNTs are functional, allowing these MNTs to be delivered into the cell nucleus.

**Figure 6:**
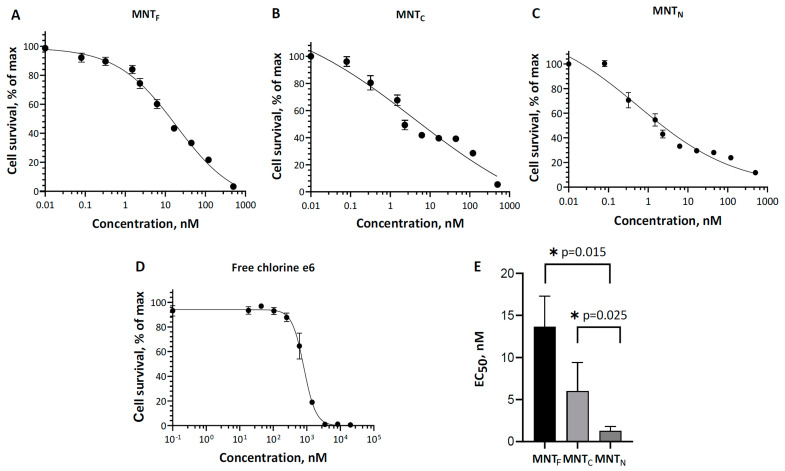
Photocytotoxicity of MNT_F_-chlorin *e*_6_ (**A**), MNTc-chlorin *e*_6_ (**B**), MNT_N_-chlorin *e*_6_ (**C**), and free chlorin *e*_6_ (**D**) on A431 cells. Experimental data of six experiments (circles) fitted using the four-parameter logistic sigmoid curves (lines) are given. EC_50_ values derived from sigmoid model approximation for each conjugate (**E**). Error bars represent standard errors (*n* = 6).

The effectiveness of the photosensitizer was assessed by the concentration of MNTs, which causes the death of 50% of cells. Figure 6 shows the results of a comparison of the effects on cells of two new MNTs and the original MNT_F_ with the attached chlorin *e*_6_. For MNT_C_, the EC_50_ was 6 ± 3 nM, and for MNT_N_, it was 1.3 ± 0.5 nM. The EC_50_ of free chlorin *e*_6_ was about 550 ± 110 nM, which shows that the truncated structures significantly enhance the photodynamic effect of chlorin e6 on A431 cells. For the original MNT_F_ with a full-size carrier module, the EC_50_ was 14 ± 4 nM; that is, the new constructs destroy cells more efficiently. At the same time, the significance of differences from the effect of the original MNT_F_ was revealed only for MNT_N_ with the N-terminal part of HMP (*p* = 0.015).

## 4. Discussion

When creating or modifying complex transport structures for intracellular delivery to target cells, it is necessary to ensure that all components of these structures retain their functionality. Due to the ligand module, which is able to bind to EGFR, MNTs are able to recognize cancer cells and penetrate them via receptor-mediated endocytosis. As previous work has shown, the K_d_ for MNT_F_ with a full-size carrier module is 29 nM [20]. In experiments on A431 cells carrying an increased number of these receptors, it was shown that truncated constructs retain the ability to bind to EGFR receptors on the cell surface, forming MNT-receptor complexes with dissociation constants of approximately 10 and 20 nM, respectively. This binding of new MNTs with EGFR suggests that they are able to successfully penetrate target cells by receptor-mediated endocytosis.

The endosomal activity of the new transporters was assessed by their ability to disrupt the integrity of lipid membranes in a model system of liposomes loaded with calcein. The membranolytic properties of MNTs reflect the release of calcein from liposomes during incubation with MNTs at different pHs. The main role in the ability of MNTs to destroy membranes is played by the translocation T domain of the diphtheria toxin, which, with a decrease in pH, is able to change its conformation and integrate into the lipid bilayer [47,48,49]. The previous research has also demonstrated HMP’s potential to attach to lipid membranes and its membranolytic activity in the pH range of 3–4. The occurrence of two peaks on the pH curve of the dependency of MNTs’ endosomolytic activity may be attributed to the joint action of two modules: one emerges as a result of HMP action, while the other is DTox [20]. According to atomic force microscopy studies of lipid membranes incubated with MNT, the combined action of HMP and DTox most likely results in the formation of ring-shaped structures on the surface of lipid membranes that contain MNTs embedded in the membrane and ensure MNT release from endosomes [20]. MNT_N_ containing only the N-terminal part of HMP exhibits decreased membranolytic action. In this regard, it can be assumed that the C-terminal fragment of HMP also plays an important role in this module’s endosomolytic activity, providing endosomolytic activity in the pH 3–4 region while also causing MNTs to be incorporated into the membrane and pores to form in the pH 5–6 region. Membranolytic activity of the globin N-terminal domain of HMP is more expected [50]. Nevertheless, the effect of MNT_N_ on lipid membranes is sufficient to lead to their destruction at a slightly acidic pH. Thus, when the contents of the endosomes are acidified, both MNTs are able to disturb the lipid bilayer.

The nucleus is an important target for many drugs as it contains DNA, damage to which, as a rule, leads to cell death [51,52,53,54]. The ability to bind to importins that allow proteins with NLS to penetrate into the nucleus through the nuclear pore, as shown by thermophoresis, indicates the possibility of successful penetration of MNTs with N- and C-terminal parts of the carrier module into the nucleus.

In this article, we also investigated how the previously discovered interaction of the MNT carrier module with Keap1 can affect the effectiveness of the photodynamic action of a photosensitizer delivered to the cell nucleus. Keap1 is an Nrf2 inhibitor that controls the expression of most proteins involved in the metabolism of ROS [55,56,57]. The displacement of Keap1 from the complex with Nrf2 can be used to activate the Nrf2 system of protection against oxidative stress [58], for example, with the help of peptides or antibodies against Keap1 [59,60,61]. For MNTs targeted at Keap1 using the anti-Keap1 monobody antibody-like molecule, it has been shown that the interaction occurs both through the anti-Keap1 monobody and through HMP [28]. Earlier, we revealed by thermophoresis assay that the K_d_ for the complex of Keap1 with MNTs containing the HMP carrier module is equal to 93 ± 15 nM [28]. However, the free affibody ligand module and the free endosomolytic module, DTox, bind to Keap1 with Kd > 1 µM, indicating low-affinity, nonspecific binding [28]. Thermophoresis has revealed that MNTs with the N-terminal part of HMP interacts with Keap1 in contrast to MNTs with the C-end of HMP (Figure 4), suggesting that HMP’s binding site to Keap1 is located in its N-terminal globin domain. The interaction of MNT_N_ with Keap1 can lead to multidirectional effects. Competition with Nrf2 could weaken the photodynamic effect of chlorin e_6_ attached to MNT_N_ by activating the synthesis of enzymes that interfere with the action of ROS. In addition, oxidation of Keap1 causes the release of PGAM5, which triggers mitochondrial autophagy and protects cells from ROS excess [62]. On the contrary, the delivery of a photosensitizer to mitochondria via the Keap1-PGAM5 interaction [35,63] can cause mitochondrial damage. Mitochondria are one of the most common intracellular targets of photosensitizers for PDT [21,64,65,66]. When the outer mitochondrial membrane is damaged, cytochrome C is released into the cytosol, which triggers apoptosis [21,67]. Our experiments show that both new MNTs possess similar affinity to the importin complex (Figure 5), but differ significantly in affinity to Keap1 (Figure 4). MNT_N_, which interacts with Keap1, increases the cytotoxicity of the attached photosensitizer more strongly than MNT_C_ (Figure 6). This is neither mitigated by a slightly lower affinity for EGFR (Figure 2) nor by a less significant membranolytic action in the slightly acidic region (Figure 3). This indicates that the additional damaging effect on mitochondria may outweigh the possible activation of anti-oxidative mechanisms in this case.

Our study shows that truncation of the HMP carrier module does not affect the functional activity of the MNTs. The MNTs with a truncated carrier module demonstrated a pronounced photodynamic effect on A431 cells. This indicates that they carry out an efficient delivery of the photosensitizer to vulnerable cell compartments. It is possible that the size of the MNTs plays a significant role in penetration into cells and cellular compartments; therefore, both MNTs with truncated HMP turned out to be more cytotoxic as carriers of photosensitizers.

The hemoglobin-like *E. coli* protein (HMP), used as a carrier module in MNTs, belongs to the flavohemoglobin group. Although it is believed that the main role of HMP in bacterial cells is to neutralize NO radicals under aerobic and anaerobic conditions [68], the processes occurring in this case are still not fully understood. HMP consists of two domains: N-terminal globin and C-terminal reductase, both of which perform their function in the process of neutralizing NO [69]. HMP has been shown to exhibit enzymatic activity in human cells; for example, it has been used to study the functions of NO signaling in mammalian cells. The expression of flavohemoglobins led to increased resistance to NO-induced cell death and weakened various NO-signaling pathways [70]. According to some reports, HMP proteins are able to protect bacteria not only from nitrosative stress but also from oxidative stress caused by ROS. A number of structural features of HMP indicate the similarity of this protein to peroxidases [70,71]. It has also been shown that mutant bacteria lacking the gene encoding HMP are less resistant to oxidative stress [72], although its overexpression leads to the accumulation of peroxide and superoxide [73]. Purified MNTs contain only a small fraction of heme, making it unlikely that HMP has enzymatic action in cells. Nonetheless, it is possible that these features of full-sized HMP can be observed in eukaryotic cells, potentially weakening the oxidative processes induced by photodynamic treatment. As both HMP domains operate together to achieve reductase activity, MNTF can be less effective than truncated MNTs.

Interaction with Keap1 at the same time, as it transpires, does not interfere with the delivery process but, on the contrary, gives MNT_N_ an advantage. It is known that Keap1 is localized, in particular, on the outer membrane of mitochondria [34,35], in connection with which the alleged interaction of MNTs with Keap1 may lead to the accumulation of a photosensitizer on the outer membrane of mitochondria. Damage to mitochondria is one of the important pathways leading to cell death under the action of photosensitizers. Thus, mitochondria-associated photosensitizers can cause photodamage of the membrane-bound protein Bcl-227, which can lead to the release of caspase activators such as cytochrome c and Smac/DIABLO, or other pro-apoptotic molecules, including apoptosis-inducing factor (AIF) [74].

Photosensitizers can cause cell death through three mechanisms: apoptosis, necrosis, and autophagy. Some photosensitizers target mitochondria specifically [15]. MNTs targeting Keap1 with an anti-Keap1 monobody have been shown to boost photosensitizer efficiency by localizing on the mitochondrial membrane [28]. Thus, an increase in the cytotoxic effect of the N-terminal HMP transporter capable of binding to Keap1 may indicate that this MNT targets the mitochondria in addition to targeting the nucleus. Further research aimed at a detailed study of the affinity of the truncated MNTs towards EGFR-positive live cells as well as their internalization and intracellular distribution via live cell imaging is being planned.

## 5. Conclusions

The aim of this work was to study the possibility of the development of functional MNT structures in smaller sizes. New MNTs generated by truncating the carrier module (HMP) in this work retained the ability to recognize target cells and enhance the cytotoxic effect of delivered photosensitizers attached to them. Moreover, the photodynamic efficacy of the photosensitizer attached to MNTs containing the N-terminal part of HMP was significantly higher than when attached to either MNTs with the C-terminal part of HMP or the original full-size MNTs. Overall, this study demonstrates that the truncation of the carrier module in MNTs does not compromise MNTs’ functionality and favors the retention of the N-terminal part of the carrier module within truncated MNTs.

## Data Availability

Data are contained within this article and its Supplementary Materials.

## Supplementary Materials

The following supporting information can be downloaded at: https://www.mdpi.com/article/10.3390/pharmaceutics16081083/s1, Figure S1: an electropherogram of truncated MNTs electrophoresed using SDS PAGE Laemmli method. 1. MNTN; 2. MNTC.
