# Supplementary material for: Optimization of a Modular Nanotransporter Design for Targeted Intracellular Delivery of Photosensitizer
Source: Pharmaceutics. 2024 Aug 18;16(8):1083. doi: 10.3390/pharmaceutics16081083 (PMC11360004; doi:10.3390/pharmaceutics16081083)
Supplement: Supplementary file 1 [file pharmaceutics-16-01083-s001.zip › pharmaceutics-3108405-supplementary.pdf]

Supplementary Materials: Optimization of a Modular Nanotransporter Design for Targeted Intracellular Delivery of Photosensitizer

Rena T. Alieva, Alexey V. Ulasov, Yuri V. Khramtsov, Tatiana A. Slastnikova, Tatiana N. Lupanova, Maria A. Gribova, Georgii P. Georgiev and Andrey A. Rosenkranz

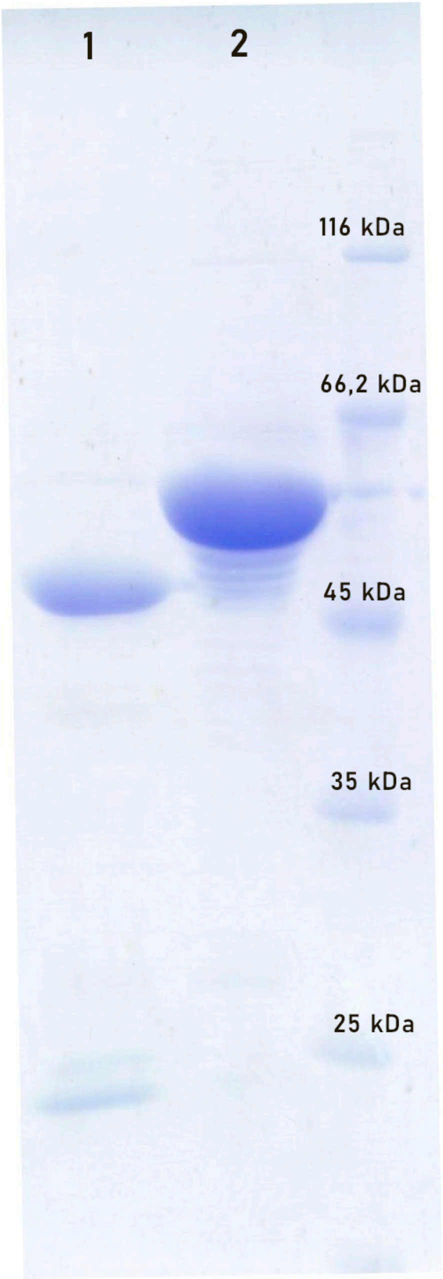

Figure S1. The figure shows an electropherogram of truncated MNTs electrophoresed using SDS PAGE Laemmli method. 1. MNT_N; 2. MNT_C.
